# Magnetoresistive sensors for measurements of DNA hybridization kinetics – effect of TINA modifications

**DOI:** 10.1038/srep41940

**Published:** 2017-02-07

**Authors:** G. Rizzi, M. Dufva, M. F. Hansen

**Affiliations:** 1 Department of Micro- and Nanotechnology, Technical University of Denmark, DTU Nanotech, Building 345B, DK-2800 Kongens Lyngby, Denmark

## Abstract

We present the use of magnetoresistive sensors integrated in a microfluidic system for real-time studies of the hybridization kinetics of DNA labeled with magnetic nanoparticles to an array of surface-tethered probes. The nanoparticles were magnetized by the magnetic field from the sensor current. A local negative reference ensured that only the specific binding signal was measured. Analysis of the real-time hybridization using a two-compartment model yielded both the association and dissociation constants *k*_on_, and *k*_off_. The effect of probe modifications with ortho-Twisted Intercalating Nucleic Acid (TINA) was studied. Such modifications have been demonstrated to increase the melting temperature of DNA hybrids in solution and are also relevant for surface-based DNA sensing. Kinetic data for DNA probes with no TINA modification or with TINA modifications at the 5′ end (1 × TINA) or at both the 5′ and 3′ ends (2 × TINA) were compared. TINA modifications were found to provide a relative decrease of *k*_off_ by a factor of 6-20 at temperatures from 57.5 °C to 60 °C. The values of *k*_on_ were generally in the range between 0.5-2 × 10^5^ M^−1^s^−1^ and showed lower values for the unmodified probe than for the TINA modified probes. The observations correlated well with measured melting temperatures of the DNA hybrids.

DNA hybridization is a key element of the majority of bioassays targeting nucleic acids. Among these, the most widespread are polymerase chain reaction (PCR) amplification and DNA microarrays. The PCR reaction starts with hybridization of a short primer sequence to the template DNA whereas the microarray recognition is based on hybridization of the target DNA to allele specific probes tethered to the surface of a microarray slide. In these applications, a high degree of hybridization translates into higher sensitivity. This can be achieved by lowering stringency to stabilize the double stranded (ds)-DNA double helix structure at the expense of a higher cross-reactivity to non-matching targets and a reduced specificity. Alternatively, ds-DNA can be stabilized by means of intercalating molecules, which have been investigated to improve hybridization assays as well as PCR amplification^1^,^2^,^3^,^4^.

A promising candidate among these is ortho-TINA (Twisted Intercalating Nucleic Acid) molecules introduced by Schneider *et al*.^5^. Insertion of ortho-TINA ((R)-1-O-[2-(1-pyrenylethynyl)-phenylmethyl] glycerol) into a oligonucleotide sequence was shown to stabilize the Watson-Crick antiparallel duplex formation^5^. The functional advantages offered by ortho-TINA modified capture probes were studied in a homogeneous hybridization assay, where the use of ortho-TINA modified probes presented a 27-fold sensitivity increase at high stringency conditions while retaining specificity to single point mutations^6^. Further, ortho-TINA modified primers for quantitative PCR (qPCR) and multiplex end-point PCR were demonstrated to provide significant advantages^7^. In these applications, primers modified with ortho-TINA at the 5′ position allowed for 100% PCR efficiency under stressed reaction conditions (high annealing temperature and low primer concentration) outperforming the unmodified counterparts.

It may be attractive to also employ ortho-TINA modifications of capture probes in surface-based DNA assays, as these may lead to a higher signal and thus a higher sensitivity with potentially less critical washing steps. However, until the present work, such studies have not been performed. Moreover, previous studies of ortho-TINA molecules have focused on demonstrating the functional advantages of the modifications but the underlying mechanics that provide the observed advantages were not investigated. Thus, there is a need for a better understanding of the hybridization kinetics of ortho-TINA modified probes.

The gold standard for measurements of surface binding kinetics is surface plasmon resonance (SPR). SPR has been applied to the real-time analysis of DNA hybridization^8^, hybridization kinetics^9^, and melting curves^10^. Wagner *et al*.^11^ used a spatial temperature gradient over an SPR substrate to assess binding kinetics simultaneously over a range of temperature conditions. SPR detects variations in the local index of refraction just above a noble metal coated surface. Thus, it is sensitive to variation in the buffer composition and temperature as well as unspecific binding.

Other techniques used for measurements of binding kinetics include real-time fluorescence^12^, quartz crystal microbalance (QCM)^13^ and surface enhanced Raman spectroscopy (SERS)^14^. Again, the main drawbacks of these techniques are the sensitivity to unspecific binding to the surface, significant cross-sensitivities to temperature and liquid properties, and, for fluorescence, a background signal from the target in solution.

Here, we apply for the first time a magnetoresistive biosensor to characterize the hybridization kinetics of magnetic nanoparticle (MNP)-labeled DNA to DNA probes tethered to a sensor surface. Magnetic biosensors provide a scalable detection method, which is insensitive to the properties of the sample matrix and which can be produced in a compact, integrated format^15^. In these, MNPs are used as labels to detect binding events via the magnetic field produced by the nanoparticles on the sensor. For example, arrays of giant magnetoresistive (GMR) sensors have been used to characterize antibody-antigen binding kinetics and the cross-reactivity of antibodies^16^. In our previous work, we have presented so-called planar Hall effect bridge (PHEB) magnetoresistive sensors and demonstrated their use for measurements on real-time melting curves of DNA hybrids to identify point mutations^17^. For the PHEB sensors, the MNPs are magnetized by the field due to the bias current passed through the sensors and thus no external electromagnets are needed. The signal of the differential sensor geometry (Fig. 1a) is given by the difference between MNPs over the top and bottom halves, respectively. Thus, when the top half of the sensor is functionalized with the detection probes and the bottom half is not functionalized, the latter functions as a local negative reference, making the sensor signal nominally insensitive to a background of MNPs in suspension^17^,^18^. Further, the sensors are integrated in a microfluidic channel using a simple click-on system and the setup includes an accurate temperature control^17^,^18^.

Here, we demonstrate that this sensor system can be used as a platform to characterize the effect of ortho-TINA molecules on the binding kinetics of a surface-based hybridization capture assay where TINA-modified capture probes are covalently linked to the surface of the magnetoresistive sensors and the biotinylated DNA target is pre-coupled to 50 nm streptavidin MNPs. Hybridization of DNA attached to MNPs at a surface differs from the reaction in solution and the use of ortho-TINA molecules in probes for surface-based sensing is here studied for the first time. We perform real-time measurements of the binding of the labeled target DNA to investigate the DNA hybridization kinetics at elevated temperatures. We extract and compare association and dissociation rates (*k*_on_ and *k*_off_) as well as melting temperatures measured for probes with and without ortho-TINA modifications. These studies provide new insight into the mechanism of action of TINA molecules and also shed light on the potential impact of employing TINA-modified probes in surface-based DNA assays.

## Theory

### Model for adsorption and desorption kinetics

**Figure 1:**
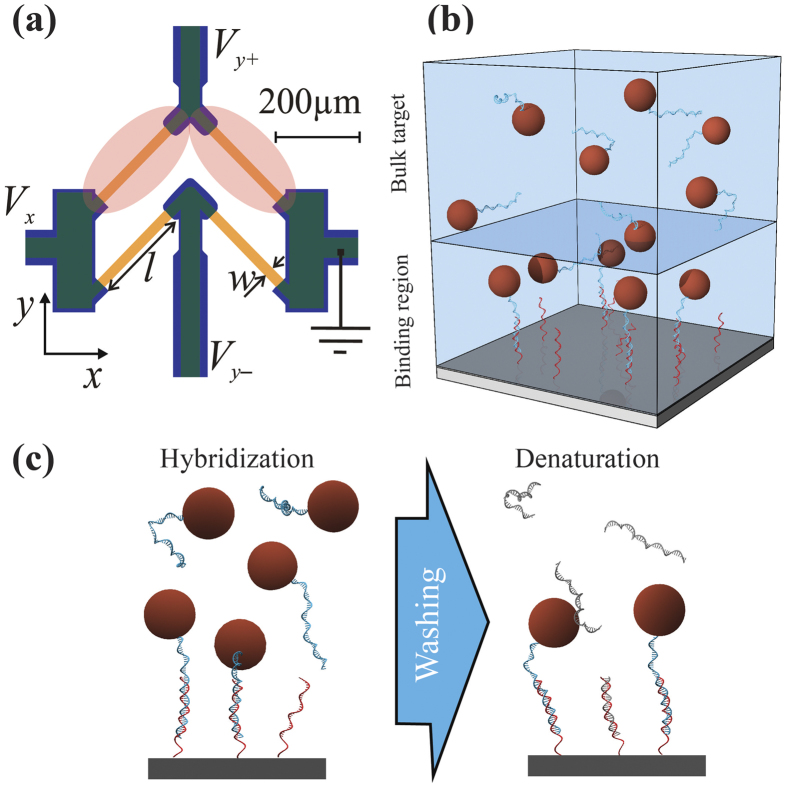
(**a**) Schematic of PHEB sensor. Orange and purple colors indicate the magnetoresistive material and contacts, respectively. The red regions indicate the areas functionalized with ss-DNA probes. **(b)** Schematic of the model for the binding kinetics. The bulk and surface volumes are indicated by boxes. Target from the bulk diffuses into the surface volume to hybridize with the surface-tethered probes. **(c)** Schematic of a hybridization-denaturation assay. In the first phase of the experiment the target (blue) binds to the sensor probes (red). After washing with a competitive target (grey), the labeled bound targets denature over time.

Since the target DNA is attached to the MNPs, the diffusion of the MNP-labeled DNA targets (MNP-targets) is limited by the MNP size, which is about 50 nm in the present study. We estimated the maximum magnetostatic force acting on the particles from the sensor stack as well as the maximum force due to the bias current applied through the sensor and found both values to be at most on the order of 1 fN. This force is so low compared to the Brownian motion of the MNPs that the deterministic motion of the particles can be neglected. To account for diffusion limitations of the magnetic labels, we use a two-compartment kinetic model to describe transport of the MNP-targets to the sensor surface^19^ (Fig. 1b); the MNP-targets diffuse from the bulk solution volume to the surface binding region where it may hybridize to the surface probes. The difference in MNP-target concentration between the two volumes drives the transport. The hybridization is modelled as a simple bi-molecular reaction between probes and MNP-targets as


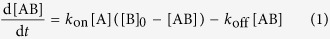



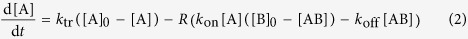


Here, [AB] is the surface density of target-probe complexes, [B]_0_ is the surface density of probes, [A] is the MNP-target concentration in the surface volume, [A]_0_ is the bulk concentration of target, *k*_on_ is the association rate, *k*_off_ is the dissociation rate, *k*_tr_ is the transport rate and *R* is a factor converting surface densities to volume densities that depends on the geometry of the system. We assume [A]_0_ to be constant during the experiment, such that the bound target is only a small fraction of the target available in solution.

In a desorption experiment, the target in solution is removed by washing ([A] = 0) and re-hybridization of the target to the surface is prevented by introducing a non-biotinylated competitive DNA target at high concentration in the wash buffer (*k*_on_ = 0) at time *t* = *t*_0_. In this limit, the desorption kinetics is described by


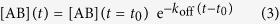


Experimentally, a desorption experiment can first be used to determine the value of *k*_off_. This value can then be used as input parameter in the adsorption kinetics in Eqs (1) and (2) for a number of target concentrations to determine *k*_on_ and the other transport parameters as described in detail in the Methods section.

## Results

**Figure 2:**
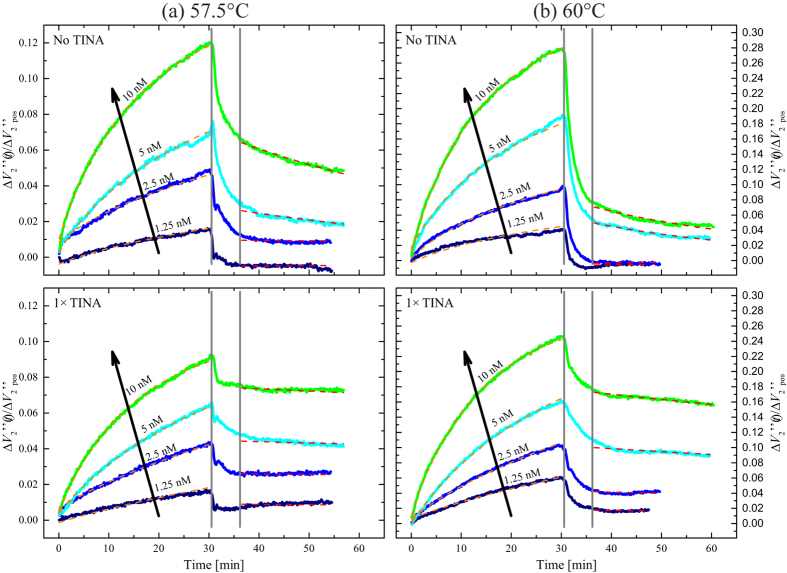
Time series of the relative signal measured during a set of hybridization and denaturation experiments at **(a)** *T* = 57.5 °C and **(b)** *T* = 60 °C. The signal was measured for the unmodified probe and for the 1 × TINA probe. Each color corresponds to a single experiment with the indicated DNA target concentration, where the signals for all probes were measured simultaneously. Dashed lines are fits to the adsorption and desorption models. The vertical lines indicate the data region excluded in the desorption fit. Plots for 2 × TINA probe and other temperatures are presented in the Supplementary information.

Experiments were performed at fixed temperature where the MNP-labeled ss-DNA target was first incubated on a single chip with three nominally identical sensors functionalized with unmodified probes, probes modified with TINA at the 5´ end (1 × TINA) and probes modified at both the 5´ and 3´ ends (2 × TINA) to study the hybridization and then washed to study the denaturation. In both cases, the liquid flow was stopped and measurements were taken with a stagnant liquid over the sensor. These measurements were repeated for four concentrations of the DNA target. Four temperatures in the vicinity of the melting temperature of the unmodified probe were investigated. A single chip was used for all experiments. Between experiments, the chip surface was regenerated using a high stringency washing. Figure 2 shows the results vs. time *t* after sample injection for the indicated target concentrations at temperatures of 57.5 °C and 60 °C for sensors functionalized with unmodified probes and 1 × TINA probes. Results obtained vs. time at the other temperatures, including those with 2 × TINA probes, are presented in the supplementary material Fig. S1.

Upon target injection at *t* = 0, the signals from both the unmodified and 1 × TINA probes were observed to increase during the 30 min of hybridization time with a consistently higher slope for higher target concentrations. At the end of the hybridization (*t* = 30 min), the signals from the unmodified and 1 × TINA probes were generally found to be of similar magnitude. At 57.5 °C (Fig. 2a), the signal from the unmodified probe was consistently higher than that from the 1 × TINA probe with the largest difference (about 30%) observed for the highest target concentration (*c* = 10 nM). At 60 °C (Fig. 2b), this difference was less pronounced (15% for *c* = 10 nM) and the signal from the unmodified probe was slightly lower than that from the 1 × TINA probe for *c* = 1.25 nM and *c* = 2.5 nM.

At *t* = *t*_0_ = 30 min, the system was washed for 1 min 20 s with washing buffer without MNPs and containing an unlabeled competitive ss-DNA target at a high concentration. Subsequently, the liquid flow was stopped and the system left with the liquid stagnant. The competitive target was introduced to inhibit re-hybridization of the sample to the surface (an example with no competitive target is given in the Supplementary Information, Fig. S2). During and immediately after washing, the signals decayed faster than exponentially. At about *t* = 35 min the signal decay slowed down and resembled the exponential decay expected from Eq. (3). At both temperatures in Fig. 2, the signal from the unmodified probe decayed faster and settled at a lower level than that from the 1 × TINA probe. This was particularly pronounced at 60 °C, where the signal from the unmodified probe at *t* = 60 min was about 25–30% of that from the 1 × TINA probe.

At each temperature, the measurements were analyzed in two steps. First, the value of *k*_off_ was obtained from the desorption data by fitting Eq. (3) to data obtained from *t* = 37 min to *t* = 60 min. Data in the time window from *t* = 30 min to *t* = 37 min (indicated by grey vertical lines in Fig. 2) were excluded in the fitting, since they could depend on the washing conditions (buffer temperature and liquid flow rate). The value of *k*_off_ was fixed in the subsequent analysis of the adsorption data. The use of the magnetic labels increased the size of the target species. Therefore, diffusion of the target to the surface probes played a significant role in the reaction kinetics. This was taken into account by use of the two-compartment adsorption model in Eq. (2) via the transport rate, *k*_tr_, which was kept as a shared parameter for all measurements performed at each temperature. The result of the fitting procedure is shown as the dashed lines in Fig. 2. The fits for the data not shown in Fig. 2 were of similar quality (Fig. S1).

**Figure 3:**
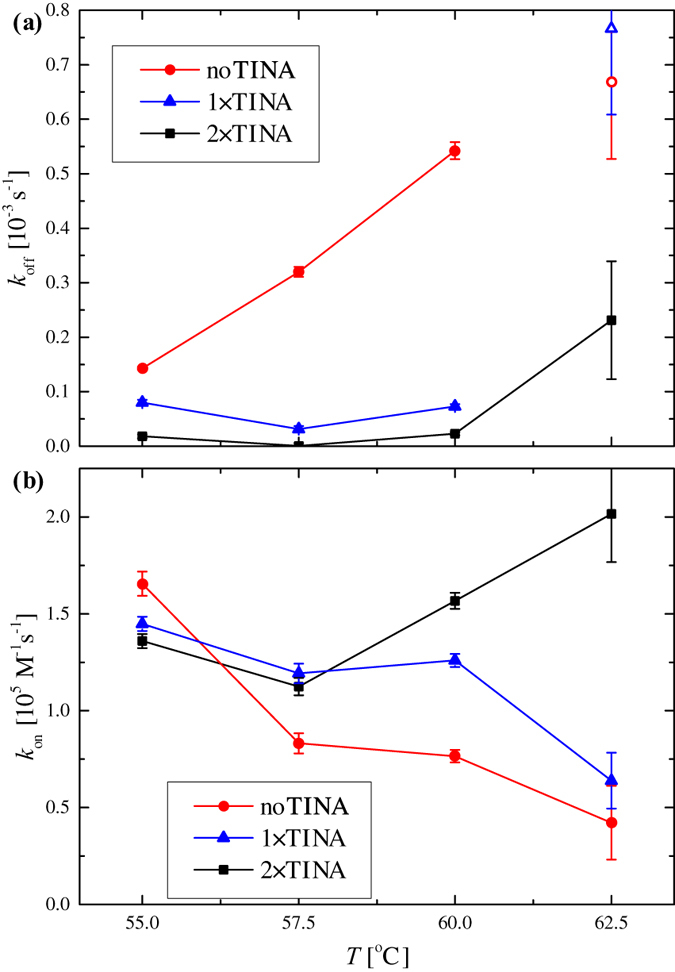
Temperature variation of parameters obtained by fitting of the kinetic model to the adsorption-desorption data for the three probes. **(a)** *k*_off_ obtained from desorption data (filled) and adsorption data (open), respectively. **(b)** *k*_on_ obtained from adsorption data with *k*_off_ fixed to the values obtained from the desorption data. Error bars are confidence intervals from the fitting routine.

Figure 3a shows the temperature dependence of *k*_off_ obtained from the analysis of the denaturation data (filled symbols) and hybridization data (open symbols). For the unmodified probe, *k*_off_ was higher than for the TINA-modified probes from *T* = 55 °C to *T* = 60 °C and increased four-fold with the increasing temperature. The TINA-modified probes on the other hand showed much lower and nearly temperature-independent values of *k*_off_ up to 60 °C. At *T* = 60 °C, the values of *k*_off_ for the TINA-modified probes were about a factor of 7-20 lower than that for the unmodified probe. Thus, the dissociation rate of the DNA hybrids was significantly reduced by the introduction of TINA molecules in the capture probe. At *T* = 62.5 °C, the denaturation was proceeding too fast to reliably fit the desorption data (Fig. S1) and the values of *k*_off_ for the unmodified and 1 × TINA probes were obtained by fitting the adsorption model with *k*_off_ as a free parameter.

Figure 3b shows the corresponding values of *k*_on_ obtained from the subsequent analysis of the adsorption data. At the lowest investigated temperature (55 °C) the values of *k*_on_ were comparable for the three probes with a slightly higher value observed for the unmodified probe. When the temperature was increased, both the unmodified and 1 × TINA probes showed values of *k*_on_ that decreased approximately linearly with temperature with lower values for the unmodified probe. At *T* = 60 °C, the 2 × TINA probe showed a significantly higher value of *k*_on_ than the two other probes. Table S1 reports all parameters obtained from the analysis of the adsorption-desorption experiments.

**Figure 4:**
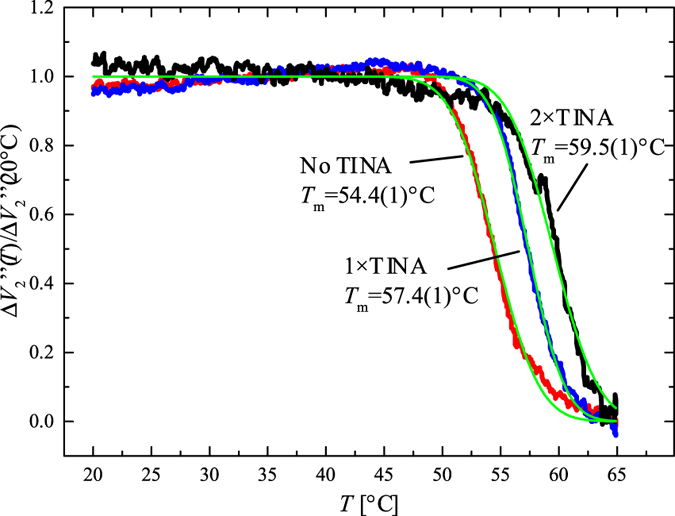
Melting curves measured on-chip for the three different probes. The three curves correspond to signal from three sensors on the same chip measured in a single experiment where the temperature was ramped from *T* = 20 °C to 65 °C at 0.1 °C/s. Signals were normalized to their initial value at *T* = 20 °C. Melting temperatures (*T*_m_) obtained by error function fits to the melting curves (in parenthesis, confidence interval on the last significant digit from the fitting routine).

We also measured the melting curves of the surface-tethered DNA hybrids. In this experiment, three sensors on the same chip were functionalized with the three different probes to measure the melting curves simultaneously. After hybridization, the sensor temperature was ramped from *T* = 20 °C to 65 °C at 0.1 °C/s. Figure 4 shows the three melting curves, normalized to their initial value at *T* = 20 °C. The curves showed a clear melting transition as expected for denaturing of DNA hybrids confirming that the MNPs were indeed tethered to the sensor surface via DNA hybridization. From error function fits of the melting curves, we obtained melting temperatures of *T*_m_ = 54.4(1) °C, 57.4(1) °C and 59.5(1) °C for the unmodified, 1 × TINA and 2 × TINA probes, respectively, where numbers in parentheses are the uncertainties on the last digit obtained from the fitting routine.

## Discussion

For the first time, we have demonstrated the use of a magnetoresistive sensor setup to measure the real-time reaction kinetics of DNA hybridization and denaturation in a microfluidic system under controlled liquid and temperature conditions. Thanks to the differential sensor design with a local reference, reliable real-time hybridization data were obtained in spite of the large background from magnetic beads in suspension over the sensor during hybridization. Further, the differential sensor design reduced possible background contributions due to unspecific binding of MNPs as these influence the two sensor parts identically^17^,^18^.

The integrated sensor design and operation further allowed for real-time investigation of the washing and subsequent isothermal denaturation of the DNA hybrids. Using the presented measurement protocol and analysis scheme in terms of the two-compartment adsorption-desorption model for a series of four target concentrations, we were able to determine robust values of *k*_off_ and *k*_on_ for the DNA target to matching DNA probes with and without ortho-TINA modifications. The diffusion limitations due to the size of the magnetic labels under the employed no-flow conditions were accounted for in the two-compartment model. The fits were generally correctly describing the data although only few parameters were free in the analysis. However, it was necessary to discard the data obtained for the first 400 s after washing, where the signal decrease was faster than exponential. We speculate that this behavior is due to the washing procedure. Both the flow rate and temperature of the washing solution may affect the kinetics and the sensor response, e.g., due to shear forces acting on the magnetic beads and/or a shift of the sensor offset due a temperature variation. Thus, there is a stabilization period that we cannot correct for, which will be subject of future investigation.

The values of *k*_on_ and *k*_off_ obtained from the analysis concur with the magnitudes for DNA hybridization reported in literature of *k*_on_ ≈ 10^5^ M^−1^ s^−1^ and *k*_off_ ≈ 10^−4^ s^−1^ (0.2 M NaCl, 20 °C, 20 bp sequence)^20^. We note that the values for surface-based sensing may differ from those in solution due to, e.g., electrostatic interactions^21^,^22^,^23^. Moreover, the MNPs may also influence the hybridization reaction, e.g., by blocking surface access to other target molecules, by electrostatic interactions or via their Brownian motion. We expect the same influence of such effects on all probes, particularly in the limit of sparse surface probes.

Compared to SPR, QCM and SERS, the presented method has the advantage that it samples on a time scale of seconds or shorter and is largely insensitive to the variation in liquid composition (including a background of labels) and temperature. Moreover, the number of magnetoresistive sensors is scalable such that many probes can be investigated in a single experiment using a simple electronic readout. The sensor chip with integrated fluidics has the potential to be fabricated at relatively low cost and with a compact form factor.

The presented method was used to analyze the effect of introducing ortho-TINA modifications on surface-tethered capture probes on the hybridization kinetics to a matching ss-DNA target labeled with MNPs. These measurements provide insight into why such modifications result in improved functional performance at elevated temperatures as observed by Schneider *et al*. under solution phase conditions^6^. Our study provides the first real-time characterization of the effect of such modifications vs. temperature for surface-tethered probes under conditions that are more similar to those employed in DNA microarrays.

We found that the inclusion of TINA modifications in the surface-tethered probes dramatically modified the hybridization and denaturation of DNA duplexes at elevated temperatures. From the measured values of *k*_off_ (Fig. 3a), we found that the TINA modifications significantly increased the stability of the target-probe assembly. The values of *k*_off_ were found to be smaller for TINA modified probes compared to the unmodified probe up to 60 °C. Moreover, we found only little temperature variation of *k*_off_ for the 2 × TINA probe in the investigated temperature range.

The analysis of the hybridization data revealed effects of TINA molecules on *k*_on_ that were not directly evident from the time-dependent data. For *T* > 57.5 °C, all TINA modified probes showed a higher *k*_on_. The value of *k*_on_ for the unmodified and 1 × TINA probes dropped with increasing temperature as expected near and above the melting temperature. Conversely, *k*_on_ measured for the 2 × TINA probe increased with increasing temperature. We speculate that the 2 × TINA modification may increase the stability of hairpins formed by short complementary sequences on the probes (2–3 bp). If so, the observed increase of *k*_on_ may be due to more probes becoming viable at elevated temperature as the hairpin structures are broken.

In the melting curve analysis, we found that the TINA modifications in the 1 × TINA and 2 × TINA probes increased the melting temperature by Δ*T*_m_ = 3 °C and 5 °C, respectively. Schneider *et al*.^6^ measured the increase in melting temperature due to TINA modified probes in a solution-based hybridization assay. They reported Δ*T*_m_ = 3.9 °C and 5.7 °C for slightly shorter (18 bp) probes modified with one or two ortho-TINA molecules at the 5′ or at both 5′ and 3′ ends, respectively. Our results for *k*_off_ may justify the observed increase in *T*_m_ upon introduction of the TINA modifications. Both the 1 × TINA and 2 × TINA probes showed a lower *k*_off_ at elevated temperatures than the unmodified probe and consequently a higher *T*_m_.

We also observed a higher value of *k*_on_ at all investigated temperatures for the TINA-modified probes. This, with the lower *k*_off_ value, allowed for a more efficient hybridization under stringent binding conditions (higher temperature). This was measured for surface-tethered probes, and is thus relevant for surface-based assays. However, a similar functional effect of TINA molecules was observed on the hybridization kinetics in solution, where Schneider *et al*.^7^ reported a high efficiency of PCR using TINA modified primers in stressed reaction conditions (high annealing temperature and low primer concentration). Both annealing temperature and primer concentration are critical to reduce cross-reactivity to non-specific targets. The increase in robustness of the reaction can simplify the design of multiplexed PCR assays and allows for the use of a higher annealing temperature. Their results are consistent with our measured temperature dependence of *k*_on_ and *k*_off_. TINA molecules stabilize DNA hybrids also in solution and a low *k*_off_ at the elongation temperature leads to a higher efficiency of PCR.

Hybridization at high temperature can be advantageous also for surface-based assays, since the higher temperature translates into faster diffusion of the target species resulting in higher sensitivity and a faster assay. This is particularly relevant when the DNA target is attached to MNPs. Moreover, the higher stability of TINA modified probes allows for the use of shorter probes to increase the difference in melting temperature caused by a mismatch and thus the specificity. Similarly, using TINA molecules it may be possible to homogenize the optimal washing stringency for a wide set of probes on a microarray with no need to extend some of the probes.

In conclusion, we have for the first time demonstrated the use of an array of magnetoresistive sensors for real-time measurements of the isothermal hybridization and denaturation kinetics of DNA target labeled with MNPs to surface-tethered DNA probes with zero to two modifications with ortho-Twisted Intercalating Nucleic Acid molecules. This work significantly expands on previous works on the functional effects of TINA molecules on bulk hybridization assays^6^ and on PCR^7^ by including the effects of the surface and the nanoparticles and it presented the first measurements of the kinetic parameters of the reaction at relevant temperatures.

The presented technology is compact as it does not rely on external electromagnets and it provides real-time data of the specific hybridization even in the presence of a large background from MNPs in suspension. The approach is scalable as a larger number of probes can be investigated by expanding the array of sensors to contain more sensors and the instrumentation can be fabricated in a compact and comparatively low-cost format. These features provide a unique advantage for such studies compared to competing technologies that show significant cross-sensitivities to temperature and liquid properties or are not easily scalable.

## Methods

### Sensors and setup

In this study we used the differential planar Hall effect (dPHEB) sensor design presented previously^17^. Briefly, each sensor (Fig. 1a) consisted of four magnetoresistive elements (*l* × *w* = 250 μm × 25 μm) arranged in a Wheatstone bridge geometry. A current passed through a sensor element generates a magnetic field, which is used to magnetize the MNPs. These produce a magnetic field resulting in a change in the resistance of the sensor element. The magnetic bead signal from this geometry is given by the difference in signals between the top and bottom halves of the bridge and thus the bottom half of the bridge functioned as a local negative reference. The magnetoresistive elements consisted of the sputter-deposited top-pinned magnetic stack Ta(15 nm)/Ni_80_Fe_20_(30 nm)/Mn_80_Ir_20_(10 nm)/ Ta(5 nm).

Electrical contacts of Ti(5 nm)/Pt(100 nm)/Au(100 nm)/Ti(5 nm) were deposited by electron beam evaporation. Each chip contained five sensors. The sensors were coated with 1000 nm of Ormocomp (micro-resist technology GmbH, Berlin, Germany) to electrically insulate the sensor from the liquid sample.

A Poly(methylmethacrylate) (PMMA) lid defined a microfluidic channel over the sensors (1 × 1 × 5 mm^3^) and established electrical contact via spring-loaded pins as described elsewhere^24^,^25^. Liquids were injected in the microfluidic system using a syringe pump. The setup further provided control of the sensor temperature with an accuracy of 0.1 °C via a custom setup with a Peltier element and an LFI-3751 temperature controller (Wavelength Electronics, Inc., MT, USA)^17^.

All sensors were voltage-biased in parallel with an AC voltage with RMS value *V*_bias = _1.8 V at a frequency *f* = 187 Hz using a high-fidelity audio amplifier. The resistance of a bridge was about 90 Ω and the sensor low-field sensitivity was *S*_0_ ≈ −300 V/(AT). The sensor bridge voltages of all sensors were measured simultaneously using Stanford Research System (SRS) SR830 lock-in amplifiers equipped with SRS SR552 pre-amplifiers. The magnetic bead signal was detected in the second harmonic out-of-phase lock-in signal, 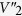, as described by Rizzi *et al*.^17^ Results are presented as the *change* in this signal, 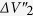, after sample injection.

### DNA probes and sensor functionalization

The magnetic sensors used for real-time studies of the DNA hybridization and denaturation kinetics were functionalized to covalently bind DNA probes with an amino modification of the 5′ end to the Ormocomp passivation layer as described elsewhere^17^. The probes were spotted on the two upper magnetoresistive elements of each sensor as depicted in Fig. 1a, while the bottom two elements served as a local negative reference. Three different probes were immobilized on three sensors on the same chip. The three probes had the same sequence (see Table 1) but presented no modification (No TINA), one ortho-TINA modification at the 3′ end (1 × TINA) or ortho-TINA modifications at both the 3′ and 5′ ends (2 × TINA). A biotinylated probe was used as direct linking for streptavidin coated particles over a fourth positive reference sensor. TINA modified probes were obtained from Eurofins MWG Synthesis GmbH (Germany). All other oligonucleotides were obtained from DNA Technology A/S (Denmark).

### DNA target and magnetic labels

The target used in the experiments (Table 1) is a 120 bp ss-DNA with a biotin modification at the 5′ end to allow for binding to streptavidin coated MNPs. A solution of target DNA in 2 × Euro-Optima PCR buffer (20.8 mM Tris-HCl, 113.6 mM Trizma-base, 32.2 mM (NH_4_)_2_SO_4_, 60 mM NaCl, 0.01% Tween80), 6 mM MgCl_2_, 0.16% nonacetylated Bovine Serum Albumin) was mixed 1:1 (v:v) to stock solution of MACS streptavidin microbeads with a diameter of 50 nm (Myltenyi Biotec Norden AB, Lund, Sweden). The initial concentration of DNA target in 2 × Euro-Optima PCR buffer was selected to obtain final concentrations of DNA *c* = 1.25 nM, 2.5 nM, 5 nM and 10 nM, respectively. The concentration of MNPs was not varied. Prior to injection, the sample was incubated at room temperature for 5 min to ensure linking of the biotinylated target DNA to the streptavidin magnetic beads.

### DNA hybridization and denaturation

The target sample (20 μL) was injected over the sensor at time *t* = 0 and incubated with no liquid flow for 30 min. We studied hybridization and denaturation at constant temperature for *T* = 55 °C, 57.5 °C, 60 °C, and 62.5 °C.

After 30 min of incubation, the sensor was washed with a 10 nM solution of ss-DNA in 1 × Euro Optima PCR buffer at a flow rate of 30 μL/s for 80 s. This ss-DNA was complementary to the probe but not biotinylated. It was employed as competitive target to prevent re-hybridization of the MNP labeled target. After washing, the signal decrease due to DNA denaturation was measured with no liquid flow for 30 min or until steady-state was reached.

Between experiments, all remaining DNA hybrids were denatured and the chip surface regenerated by washing with MilliQ water at *T* = 70 °C and drying with N_2_.

### Data correction and adsorption-desorption analysis

The signal due to MNPs bound on the sensor surface is reported as the variation 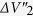 of the second harmonic out-of-phase signal upon injection of the sample. An offset due to buffer injection over the chip was measured separately, without magnetic particles and subtracted from 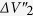. To correct for the temperature dependence of the sensor sensitivity, all signals were normalized with the saturation signal 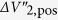 from the positive reference sensor that was functionalized directly with biotinylated DNA and directly bound the streptavidin MNPs^18^. The signal was filtered and the number of data points reduced by data binning in 7 s intervals.

To perform the analysis, the desorption model, Eq. (3), was first fitted to the relative signal measured after washing. Washing with the room temperature washing buffer reduced the sensor temperature. After 80 s of washing, the flow was stopped and the temperature stabilized. To exclude possible effects of temperature variation introduced by the liquid exchange, the first 400 s after washing were discarded. One set of experiments comprised measurements with the four indicated DNA target concentrations for a single target on sensors functionalized with unmodified, 1 × TINA and 2 × TINA probes at a single fixed temperature. The desorption model was fitted with *k*_off_ as a free and shared parameter for all four curves in the data set and with [AB]_0_ as a free parameter for each curve in the data set. Subsequently, the adsorption model, Eqs. (1)-(2), 2, was fitted to the relative signals measured for the four target concentrations during hybridization at fixed temperature. At each temperature, one dataset comprised measurements at four concentrations for each of the three probes. The model was fitted simultaneously to all the curves with shared fitting parameters. The following parameter bindings were used in the fitting: For each probe and varying target concentration, *k*_on_ and [B]_0_ were free fitting parameters and *k*_off_ was fixed to the value obtained from the desorption experiments. The values of *k*_tr_ and *R* were free fitting parameters shared between all probes and target concentrations. Additionally, an offset was allowed for each curve. Due to high dissociation rate, it was not possible to fit the desorption model to the measurement at 62.5 °C of No TINA and 1 × TINA probes. For these data, *k*_off_ was obtained from fitting to the adsorption data as described in the supplementary information.

### On-chip melting curve

Melting profiles of target bound to the three different DNA probes were measured as described previously^18^. After 30 min of hybridization of 5 nM of target DNA in 2 × SSC at 50 °C, the sensor was flushed with 0.05 × SSC at 20 °C and left with no liquid flow. The magnetic signal was measured while sweeping the temperature from 20 °C to 65 °C at a rate of 0.1 °C/s. The sensor signal was corrected for temperature dependence and the melting temperatures for the different probes were obtained by fitting of the error function as detailed in Rizzi *et al*.^18^.

## Additional Information

**How to cite this article**: Rizzi, G. *et al*. Magnetoresistive sensors for measurements of DNA hybridization kinetics – effect of TINA modifications. *Sci. Rep.* **7**, 41940; doi: 10.1038/srep41940 (2017).

**Publisher's note:** Springer Nature remains neutral with regard to jurisdictional claims in published maps and institutional affiliations.

## Supplementary Material

Supplementary Information

## Figures and Tables

**Table 1:** Sequences of probes and targets used in this work. **Y** is used to mark the location of ortho-TINA molecules.

Name	Sequence
Positive ref.	NH2-C6-5′-(9 × T)TGC GAG CTT CGT ATT ATG GCG -3′-TEG-Biotin
No TINA	NH2-C6-5′-(9 × T)GAG GAG AAG TCT GCC GTT ACT G-3′
1 × TINA	NH2-C6-5′-(9 × T)**Y**GAG GAG AAG TCT GCC GTT ACT G-3′
2 × TINA	NH2-C6-5′-(9 × T)**Y**GAG GAG AAG TCT GCC GTT ACT G**Y**-3′
Target	Biotin-TEG-5′-[…]CAG TAA CGG CAG ACT TCT CCT C […]-3′
