# Supplementary material for: Magnetoresistive sensors for measurements of DNA hybridization kinetics – effect of TINA modifications
Source: Sci Rep. 2017 Feb 7;7:41940. doi: 10.1038/srep41940 (PMC5294463; doi:10.1038/srep41940)
Supplement: Supplementary Information [file srep41940-s1.pdf]

Magnetoresistive sensors for measurements of DNA hybridization kinetics – effect of TINA modifications

G. Rizzi,[a] M. Dufva[a] and M. F. Hansen[a*]

[a]Department of Micro- and Nanotechnology, Technical University of Denmark, DTU Nanotech, Building 345B, DK-2800 Kongens Lyngby, Denmark.

*Corresponding Author: ph: (+45) 4525 6338, E-mail: Mikkel.Hansen@nanotech.dtu.dk

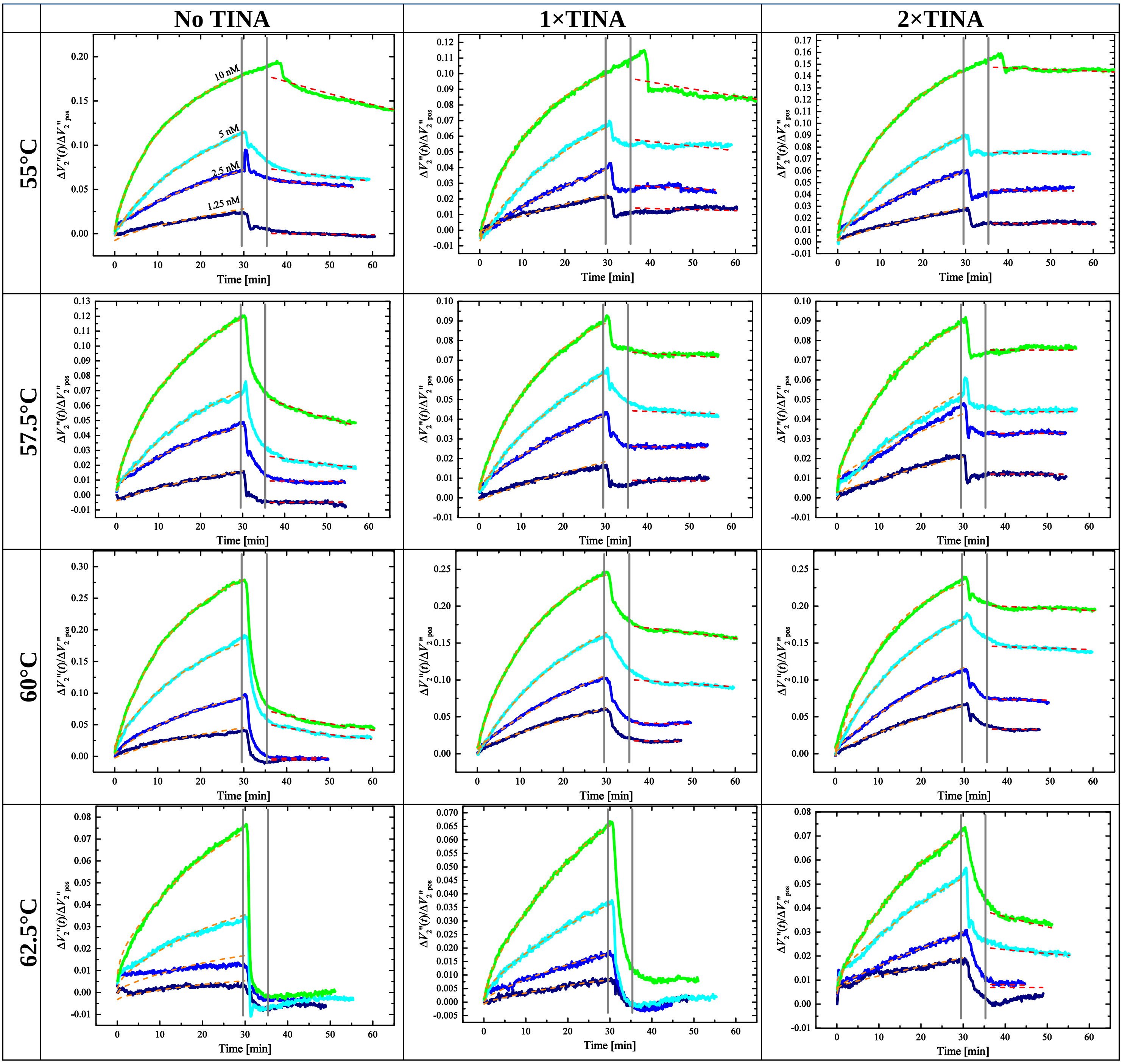

Figure S1: Time series of all adsorption-desorption experiments. The tested target concentrations follow the order from the top-left corner graph. The dashed lines are the fits of the kinetic model to the data. The vertical lines mark the data excluded from the desorption fits.

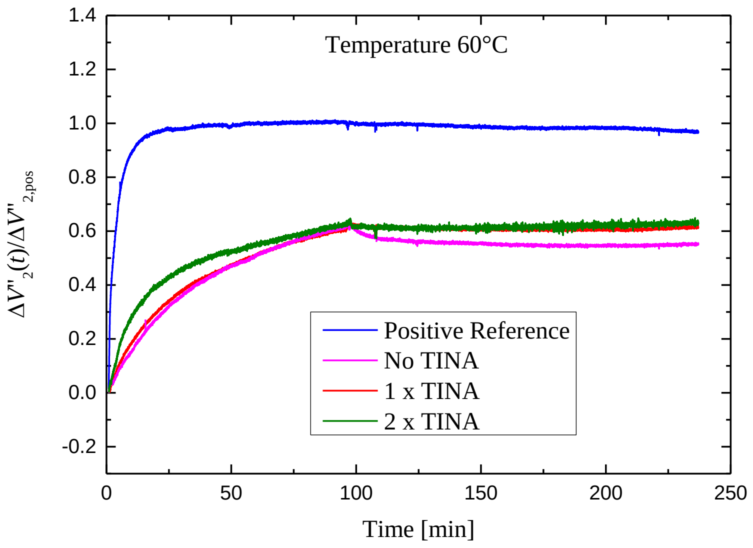

Figure S2: Time series of absorption-desorption of 10 nM target at 60°C without competing DNA target. After 100 min binding time the signals from the three probes approached saturation at the same signal level. After washing without competing free DNA target, the signal from the No TINA probe decreased very slowly. The signals from the other probes did not vary. Without the competing free DNA, the desorption was slow or non-existing. We hypothesize that, due to the slow particle diffusion, denatured DNA had a high probability to re-hybridize to the surface probes.

Table S1: Fitting parameter obtained from all the kinetic model fits. The values of k_{off} were obtained from fits to the desorption model and used as parameter for the adsorption model fit. Cells highlighted in green are the parameters shared between the different probes for experiments run at the same temperature. At 62.5°C, it was possible to fit the desorption model only for the 2×TINA probe. Thus, at this temperature, the values of k_{off} for the No TINA and 2×TINA probes (in blue) were free fitting parameters in the adsorption model. Furthermore, it was necessary to bind $[B]_0$ for the No TINA probe (in red) to 0.5. Numbers in parentheses indicate the error on the last digit obtained from the fitting procedure.

**No TINA**					
T **[°C]**	k_{off} [10^{-4} s^{-1}]	k_{on} [10^{5} $M^{-1}s^{-1}$]	k_{tr} [10^{-3} s^{-1}]	$[B]_0$ [10^{-1}]	*R* [10^{-8} M]
**55**	1.45(5)	1.66(6)	1.5(1)	2.11(2)	5.6(4)
**57.5**	3.2(1)	0.83(5)	1.6(4)	1.84(5)	4(1)
**60**	5.4(1)	0.76(3)	1.7(2)	5.4(1)	1.6(3)
**62.5**	6(1)	0.4(2)	6.4(8)	5	70(8)
**1×TINA**					
T **[°C]**	k_{off} [10^{-4} s^{-1}]	k_{on} [10^{5} $M^{-1}s^{-1}$]	k_{tr} [10^{-3} s^{-1}]	$[B]_0$ [10^{-1}]	*R* [10^{-8} M]
**55**	0.80(5)	1.45(4)	1.5(1)	1.22(1)	5.6(4)
**57.5**	0.31(5)	1.19(5)	1.6(4)	1.00(1)	4(1)
**60**	0.73(4)	1.26(3)	1.7(2)	2.78(1)	1.6(3)
**62.5**	7(1)	0.6(1)	6.4(8)	0.22(4)	70(8)
**2×TINA**					
T **[°C]**	k_{off} [10^{-4} s^{-1}]	k_{on} [10^{5} $M^{-1}s^{-1}$]	k_{tr} [10^{-3} s^{-1}]	$[B]_0$ [10^{-1}]	*R* [10^{-8} M]
**55**	0.18(6)	1.35(5)	1.5(1)	1.54(2)	5.6(4)
**57.5**	0.01(5)	1.12(4)	1.6(4)	0.93(1)	4(1)
**60**	0.23(2)	1.57(4)	1.7(2)	2.55(1)	1.6(3)
**62.5**	2(1)	2.0(2)	6.4(8)	8.2(2)	70(8)
